# Editorial: Comorbidity in bipolar disorder and schizophrenia, volume III

**DOI:** 10.3389/fpsyt.2023.1356817

**Published:** 2024-01-09

**Authors:** Giovanni Martinotti, Michele Fornaro, Domenico De Berardis

**Affiliations:** ^1^Department of Neurosciences, Imaging and Clinical Sciences, Università degli Studi G. D'Annunzio, Chieti, Italy; ^2^Psychopharmacology, Drug Misuse and Novel Psychoactive Substances Research Unit, School of Life and Medical Sciences, University of Hertfordshire, Hatfield, United Kingdom; ^3^Clinical Section of Psychiatry and Psychology, Department of Neuroscience, Reproductive Sciences, and Odontostomatology, University School of Medicine Federico II, Naples, Italy; ^4^Department of Psychiatry, Azienda Sanitaria Locale 4, Teramo, Italy; ^5^School of Nursing, University of L'Aquila, L'Aquila, Italy; ^6^International Centre for Education and Research in Neuropsychiatry, Samara State Medical University, Samara, Russia

**Keywords:** bipolar (affective/mood) disorders, schizophrenia, comorbidity, recovery, treatment, prognosis factors, anxiety, substance use disorder

Understanding comorbidities in bipolar disorder and schizophrenia is crucial for providing comprehensive and effective care for individuals affected by these complex psychiatric conditions (1, 2). Comorbidities refer to the presence of two or more distinct disorders or conditions within the same individual, and the co-occurrence of bipolar disorder and schizophrenia with other mental health disorders, physical health conditions, and substance use disorders is well-documented. This Research Topic aimed to explore the complexities of comorbidities in bipolar disorder and schizophrenia, including their impact on diagnosis, treatment, and clinical outcomes.

Comorbidities in bipolar disorder and schizophrenia encompass a wide range of psychiatric and medical conditions that can significantly influence the course of illness and treatment outcomes. Common comorbid psychiatric disorders include anxiety disorders, personality disorders, substance use disorders, and attention-deficit/hyperactivity disorder (ADHD), among others (3–5). Individuals with bipolar disorder or schizophrenia may also experience comorbid medical conditions such as cardiovascular disease, diabetes, and obesity, which can further complicate their overall health and wellbeing. The presence of comorbid psychiatric disorders in individuals with bipolar disorder or schizophrenia has important implications for diagnosis and treatment. The overlapping symptoms and shared risk factors between these conditions can pose challenges for accurate differential diagnosis, potentially leading to delayed or misdiagnoses. For example, symptoms of anxiety or substance use disorders can intersect with the mood and psychotic symptoms of bipolar disorder and schizophrenia, complicating the identification of primary and comorbid conditions.

Moreover, the presence of comorbid psychiatric disorders can impact the choice and management of treatment for individuals with bipolar disorder or schizophrenia (4, 6, 7). Co-occurring anxiety disorders, for example, may require integrated treatment approaches that address both anxiety and mood or psychotic symptoms. Similarly, substance use disorders can complicate medication adherence and contribute to increased risk of relapse or exacerbation of psychiatric symptoms. In addition to psychiatric comorbidities, individuals with bipolar disorder and schizophrenia often face a greater burden of physical health comorbidities compared to the general population. Medical conditions such as cardiovascular disease, diabetes, and obesity are disproportionately prevalent among individuals with severe mental illness, contributing to reduced life expectancy and poorer physical health outcomes. The interplay between psychiatric and physical health comorbidities underscores the importance of comprehensive and integrated care that addresses the diverse needs of individuals affected by bipolar disorder and schizophrenia.

Comorbidities in bipolar disorder and schizophrenia also present challenges for treatment adherence and engagement in care (8–10). The complex interplay between multiple comorbid conditions can contribute to treatment resistance and greater difficulty in achieving stabilization of mood and psychotic symptoms. Individuals with comorbidities may also face greater barriers to accessing and utilizing mental health services, further exacerbating their clinical needs and functional impairments. In clinical practice, addressing comorbidities in bipolar disorder and schizophrenia requires a holistic and multidisciplinary approach that considers the diverse and interconnected needs of affected individuals. Collaborative efforts between mental health professionals, primary care providers, and specialists in relevant medical fields are essential for conducting comprehensive assessments, developing integrated treatment plans, and facilitating coordinated care that addresses both psychiatric and physical health comorbidities. The high prevalence of comorbid substance use disorders in individuals with bipolar disorder and schizophrenia presents unique challenges for treatment and recovery (11, 12). Substance use can exacerbate the severity of mood and psychotic symptoms, interfere with medication adherence, and contribute to increased risk of relapse and hospitalization. Integrated treatment models that combine psychosocial interventions with specialized substance use disorder treatment are essential for addressing the complex needs of individuals with comorbid bipolar disorder, schizophrenia, and substance use disorders.

In addition to substance use disorders, comorbid anxiety disorders are common among individuals with bipolar disorder and schizophrenia and can significantly impact their clinical presentation and quality of life (13). The presence of anxiety symptoms can exacerbate the severity of mood and psychotic symptoms, contribute to functional impairment, and increase the risk of suicide in individuals affected by bipolar disorder or schizophrenia. Integrating evidence-based treatments for anxiety disorders into the overall care plan is essential for addressing the diverse needs of individuals with comorbid anxiety and severe mental illness. The management of comorbidities in bipolar disorder and schizophrenia also necessitates a focus on addressing the physical health needs of affected individuals. The elevated risk of cardiovascular disease, diabetes, and obesity among individuals with severe mental illness requires proactive screening, prevention, and management strategies to mitigate the impact of these comorbid conditions on overall health and wellbeing. Integrated care models that encompass both psychiatric and physical health services are essential for promoting holistic and comprehensive care for individuals affected by bipolar disorder and schizophrenia comorbidities.

Research into the comorbidities of bipolar disorder and schizophrenia is critical for advancing our understanding of the complex interactions between psychiatric and medical conditions and informing the development of targeted interventions (11, 14, 15). Longitudinal studies that examine the impact of comorbidities on clinical outcomes, treatment response, and healthcare utilization can provide valuable insights into the challenges and opportunities associated with addressing the diverse needs of individuals affected by bipolar disorder and schizophrenia comorbidities. Public health initiatives aimed at raising awareness about the comorbidities in bipolar disorder and schizophrenia are essential for reducing stigma, promoting early detection, and improving access to integrated care. Greater public awareness and advocacy can contribute to enhanced support and resources for individuals and families affected by comorbid conditions, fostering a more informed and compassionate societal response to the complex challenges posed by the intersection of bipolar disorder, schizophrenia, and comorbidities.

In the last published present of this Research Topic, van Dee et al. aimed to evaluate the role of psychiatric comorbidity as a predictor of schizophrenia spectrum disorders outcomes. This research contributed to the progress of personalized prognostic prediction models by introducing a new machine learning pipeline that might enable more precise and tailored treatment for patients with schizophrenia spectrum disorders. Using such complex machine learning models and a counterfactual model explanation technique, the Authors found that at the group level, most psychiatric comorbidities had a negative influence on the predicted likelihood of symptomatic remission in schizophrenia spectrum disorders. At the individual level, the Researchers found high variability in the influence of the presence of comorbidities on the chance of remission. Thus, the results emphasized the importance of identifying and including relevant comorbidities in prediction models and provided valuable insights for improving the treatment and prognosis of individual patients with schizophrenia spectrum disorders.

Dragasek et al. analyzed data for a nationwide multicenter observational study (COSMOS, COmorbiditieS in MOst severe neurology and psychiatric disorders in Slovakia) in order to observe, measure and characterize the cross-sectional connections of psychiatric and physical comorbidities with selected demographic and clinical characteristics in patients with bipolar disorder. The results suggested that the comorbid disorders in bipolar disorder are associated with several indices of harmful dysfunction, decrements in functional outcomes, increased utilization of medical services, and can negatively affect the course of bipolar disorder, severity of bipolar disorder and its treatment. Finally, bipolar disorder comorbidities can lead to even greater disability and mortality and the findings revealed that the associations between psychiatric and somatic comorbidities of bipolar disorder seem to be bidirectional.

On the other hand, Grunze et al. performed a narrative review aimed to overview the current knowledge- and lack of- about epidemiology, physical and mental health impact and treatment of tobacco use disorder and nicotine dependence in people with bipolar disorders. They pointed out that lifetime and point prevalence of smoking in people with bipolar disorders was in the range of 45%−70% and thus about 2–3 times more frequent in such patients than in general population. Smoking, tobacco use disorder and nicotine dependence might have a harmful impact both on mental and physical health as well as mortality in people with bipolar disorders. Besides, pharmacological treatments normally follow the individual guidance for each disorder, but community-based psychosocial interventions for tobacco use disorder and nicotine dependence seemed to be suitable in people with bipolar disorders, too, as well as Cognitive Behavioral or Acceptance and Commitment based psychotherapies.

In the first published article of the Research Topic, Ferracuti et al. theorized that patients affected by schizophrenia might show peculiar gyrification of certain cortical brain areas correlated with the symptom severity, evaluating a sample of 36 patients (with a mean age of 26.5 ± 7.4 years) affected by schizophrenia undergoing structural magnetic resonance imaging to calculate the local gyrification index (LGI). They found that acute inpatients with schizophrenia showed increased LGI correlated to symptom severity in bilateral frontal, cingulate, parietal, temporal cortices, and right occipital cortex. The comparison between low hostility symptoms and high hostility symptoms showed in the first group a significantly lower LGI related to the severity of symptoms in the bilateral frontal and temporal cortices. These changes in LGI involved abnormalities in areas that have been already associated with a lack of insight and awareness of the illness, severity of psychotic symptoms, and aggressive and antisocial behavior, which are often related to involuntary hospitalization.

In conclusion, comorbidities in bipolar disorder and schizophrenia encompass a wide range of psychiatric and physical health conditions that significantly influence the clinical presentation, treatment, and outcomes of affected individuals. Addressing comorbidities requires a comprehensive and integrated approach that considers the intersecting needs of individuals with complex psychiatric and medical conditions. By fostering collaborative research, clinical practice, and public awareness initiatives, it is possible to improve our understanding of the diverse needs of individuals affected by bipolar disorder and schizophrenia comorbidities and enhance the quality of care provided to these populations.

## Author contributions

GM: Conceptualization, Data curation, Formal analysis, Funding acquisition, Investigation, Methodology, Project administration, Resources, Software, Supervision, Validation, Visualization, Writing – original draft, Writing – review & editing. MF: Conceptualization, Data curation, Formal analysis, Funding acquisition, Investigation, Methodology, Project administration, Resources, Software, Supervision, Validation, Visualization, Writing – original draft, Writing – review & editing. DD: Conceptualization, Data curation, Formal analysis, Funding acquisition, Investigation, Methodology, Project administration, Resources, Software, Supervision, Validation, Visualization, Writing – original draft, Writing – review & editing.
